# Notch-Mediated Cell Adhesion

**DOI:** 10.3390/biology5010005

**Published:** 2016-01-16

**Authors:** Akihiko Murata, Shin-Ichi Hayashi

**Affiliations:** Department of Molecular and Cellular Biology, Division of Immunology, School of Life Science, Faculty of Medicine, Tottori University, Yonago, Tottori 683-8503, Japan; shayashi@med.tottori-u.ac.jp

**Keywords:** Notch, Delta/Delta-like, Serrate/Jagged, cell adhesion, metazoan evolution

## Abstract

Notch family members are generally recognized as signaling molecules that control various cellular responses in metazoan organisms. Early fly studies and our mammalian studies demonstrated that Notch family members are also cell adhesion molecules; however, information on the physiological roles of this function and its origin is limited. In this review, we discuss the potential present and ancestral roles of Notch-mediated cell adhesion in order to explore its origin and the initial roles of Notch family members dating back to metazoan evolution. We hypothesize that Notch family members may have initially emerged as cell adhesion molecules in order to mediate multicellularity in the last common ancestor of metazoan organisms.

## 1. Introduction

Beginning with the mutant fly found by T. H. Morgan’s group in 1913 [1], and through to the cloning of the responsible gene by S. Artavanis-Tsakonas’s group in the 1980s [2,3,4], Notch family members are now recognized as essential signaling molecules that control a diverse array of cellular responses ranging from normal development to the maintenance of homeostasis in metazoan organisms. Many complex Notch signaling components are conserved in extant metazoans that are now being examined [5,6,7]; however, due to the diversified roles of Notch signaling, difficulties are associated with determining the initial roles of newly emerged Notch family members in early metazoan ancestors. When viewed from the perspective of signaling functions, the answer to this may be the establishment of different cellular statuses through the regulation of cell fate determination and differentiation between neighboring cells using a well-known mechanism called lateral inhibition [8,9,10,11].

Notch family members were initially identified as cell adhesion molecules in early fly studies [12,13]. We recently showed that the cell adhesion function was evolutionally conserved in mammalian Notch family members [14,15]. Notwithstanding the long history of this field of research, the function of cell adhesion has not received much attention and, thus, the physiological roles of Notch-mediated cell adhesion remain largely unknown, even in the fly. Since cell adhesion is the simplest function of a receptor-ligand interaction and a functional Notch signaling pathway requires many molecular components, the adhesion function of Notch family members may predate their signaling function. When did the adhesion function emerge and what was the selective advantage during the metazoan evolution?

The purpose of this review is to explore the origin of Notch-mediated cell adhesion and the initial role of Notch family members in our ancestors in light of cell adhesion. We started by introducing the fly *Drosophila* *melanogaster* and our mammalian studies showing Notch-mediated cell adhesion.

## 2. Notch, DSL Ligands, and Notch Signaling

**Figure 1:**
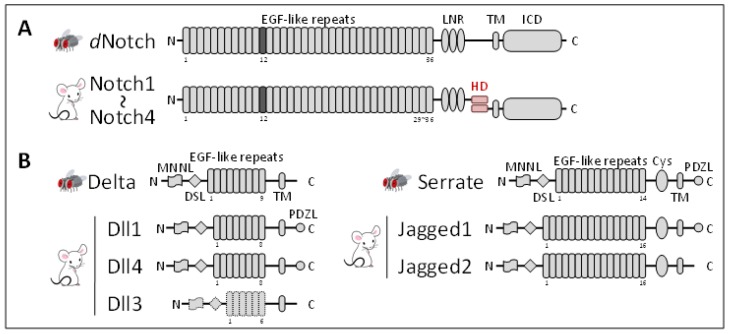
Notch family members in flies and mammals. (**A**) Flies have one Notch receptor, while mammals have four (Notch1-Notch4) with different numbers (36, 36, 34, and 29, respectively) of EGF repeats [16,17,18,19,20,21,22,23]. Most of mammalian Notch receptors are expressed as furin-processed heterodimers, while most surface *d*Notch is the unprocessed full-length form [29,30,31,32]. The core ligand-binding site of *d*Notch and mammalian Notch1 is located within the 12th EGF repeat [41], while other EGF repeats also contribute to ligand binding [13,42,43,44,45,46]. Many EGF repeats, including the 12th, bind to Ca^2+^ ions, which are critical for protein structures and ligand binding [47,48,49]. Notch receptors form a dimer, or even a multimer, on the cell surface [50,51,52]. The ICD contains several domains that mediate nuclear translocation, interactions with CSL (*i.e.*, Ankyrin repeats), and transcriptional activity (not depicted). LNR, Lin12-Notch repeats, HD, heterodimerization domain, and TM, transmembrane domain; (**B**) DSL ligands have a conserved N-terminal MNNL (module at the N-terminus of Notch ligands) domain, followed by a DSL domain and multiple tandem EGF-like repeats. MNNL, DSL, and the first three EGF repeats are all required for receptor binding [45,53,54,55,56,57,58]; however, other domains may also contribute to receptor binding [55,59]. Serrate is distinguishable from Delta by its larger number of EGF repeats and the presence of an additional cysteine-rich region (Cys). Some ligands have an intracellular PDZL (PSD-95/Dlg/Zo-1-ligand) motif that mediates interaction with PDZ-containing scaffold/adaptor proteins [60].

Our understanding of Notch family members mostly comes from the fly *D.* *melanogaster* and mammalian studies (reviewed in [16,17,18,19,20,21,22,23]). *D.* *melanogaster* possesses a single Notch receptor gene (*d*Notch) that encodes a single-pass transmembrane protein with a large extracellular domain (ECD) containing tandem epidermal growth factor (EGF)-like repeats, which are responsible for ligand binding (Figure 1A). The EGF repeats are followed by several domains that regulate the cleavage or function of Notch [24]. In contrast, mammals (rodents and human) have four paralogues of Notch receptors (Notch1-Notch4), following the two rounds of whole genome duplication that occurred during vertebrate evolution [25,26,27,28]. Most of mammalian Notch receptors are expressed on the cell surface as a processed heterodimer after cleavage by a furin-like convertase at the heterodimerization domain in the trans-Golgi network [29,30], while most surface *d*Notch is the unprocessed full-length form; however, furin-like convertase has been detected in flies [31,32] (Figure 1A).

Two Notch ligands, Delta [33] and Serrate [34], have been identified in the fly, both of which are characterized by the presence of a conserved DSL (Delta, Serrate, and Lag2) domain (thus called DSL ligands), followed by multiple tandem EGF-like repeats (Figure 1B). The five mammalian DSL ligands have been classified into the Delta-like (Dll) family (Dll1, Dll3, and Dll4) and Jagged family (Jagged1 and Jagged2) based on homology to *Drosophila* Delta and Serrate, respectively. Mammalian DSL ligands, except for Dll3, are considered to bind and activate any of the four Notch receptors [35,36,37,38,39]. Dll3 is a divergent ligand that cannot bind to or activate Notch receptors on neighboring cells [38,39,40].

**Figure 2:**
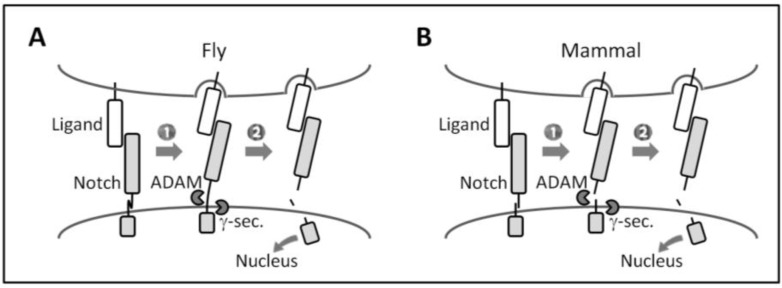
Canonical Notch signaling. (**A** and **B**) Canonical Notch signaling requires the successive cleavage of Notch by ADAMs and the γ-secretase complex. (1) Upon ligand binding, endocytosis in ligand-expressing cells is proposed to generate a pulling force that induces (**A**) conformational changes in *d*Notch ECD or (**B**) the dissociation of heterodimerized mammalian Notch ECD [24,65,66,67]; (2) This allows ADAMs to access the cleavage site of Notch ECD remaining on the membrane [24]. After γ-secretase processing, the released Notch ICD induces transcription of target genes.

The activation of canonical Notch signaling relies on direct cell-cell contact because of its transmembrane nature, and requires the endocytic activity of ligand-expressing cells and repeated proteolytic cleavage (Figure 2). Notch receptor-ligand binding between neighboring cells (*i.e.*, *trans*-interaction) induces a conformational change in (flies) or the dissociation (mammals) of Notch ECD that allows for the successive cleavage of the receptor by ADAMs (a disintegrin and metalloproteases) and the γ-secretase complex at the ECD and transmembrane domain, respectively. This permits the Notch intracellular domain (ICD) to translocate into the nucleus, in which it forms a transcriptional complex with the DNA-binding protein CSL (CBF1, Suppressor of Hairless, Lag1) (also known as RBP-Jκ in mammals) and other co-factors in order to induce the transcription of Notch target genes. Recent findings suggest the existence of a non-canonical Notch signaling pathway that is independent of CSL or γ-secretase activity [61,62,63,64].

## 3. Notch Family Members are Cell Adhesion Molecules

### 3.1. Early Fly Studies

A direct interaction between Notch and DSL ligands was initially demonstrated by cell aggregation assays with normally non-adhesive *Drosophila* Schneider’s 2 (S2) cultured cells [12,13]. S2 cells overexpressing *d*Notch (S2-*d*Notch) formed cell aggregates when cultured with S2 cells overexpressing Delta (S2-Delta) or Serrate (S2-Serrate). Cell aggregation depended on extracellular Ca^2+^ and was preserved even with the arrest of cell metabolism. Moreover, a series of Notch deletion mutants revealed that Notch ICD is not required and the 11th and 12th EGF repeats from the N-terminal end of Notch ECD are necessary and sufficient for aggregation with S2-Delta or S2-Serrate cells [13].

These early findings clearly demonstrated that cell aggregation was not a consequence of the activation of Notch signaling and was accounted for by the Notch receptor-ligand interaction itself, indicating that Notch and DSL ligands in the fly function as cell adhesion molecules. The aggregation assay was also later widely employed to show the strong adhesive force between Notch and Delta [68], and the EGF repeats required for Notch receptor-ligand binding [41,55,56,57].

### 3.2. Vertebrate Studies

**Figure 3:**
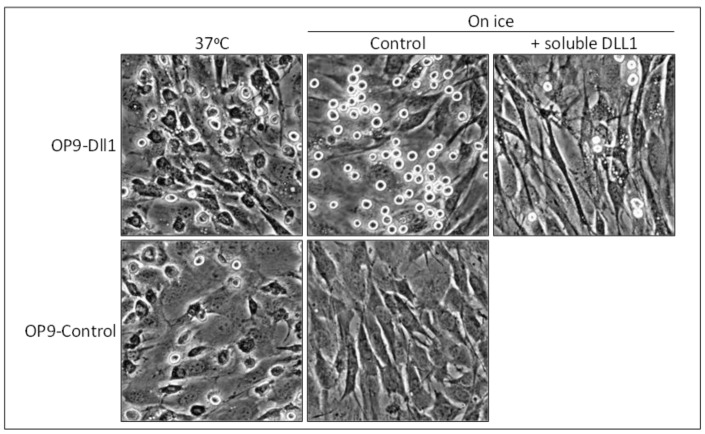
Mammalian Notch family members function as adhesion molecules. Representative photomicrographs of adherent mast cells after the removal of non-adherent cells are shown. Many adherent mast cells spread on stromal cells with a deformed morphology at 37 °C, while those on OP9-Dll1 cultured on ice tethered, maintaining their original morphology. Photomicrographs are from reference [14] (*Copyright 2010. The American Association of Immunologists, Inc.*, Bethesda, MD, USA).

We recently demonstrated that the function of cell adhesion is conserved in mammalian Notch family members [14,15]. Our first objective was to determine the role of Notch signaling in mammalian mast cells, a hematopoietic cell lineage that is best known for its roles in the mediation of allergic inflammation [69]. Notch receptors are widely expressed among mammalian immune cells, including mast cells, and control their differentiation and functions [70,71,72]. We cultured mouse mast cells [73] on stromal cells transduced with the mouse *Dll1* gene (OP9-Dll1) [74], which is the closest mammalian relative to the fly Delta [75]. We found that the adhesion of mast cells to stromal cells was promoted more on OP9-Dll1 than on OP9-Control cells (Figure 3) [14]. Moreover, mast cell adhesion on OP9-Dll1 reached its maximum very rapidly (within 15 min).

Canonical Notch signaling has been suggested to enhance cell-cell adhesion by inducing the expression and activation of cell adhesion molecules such as integrins [76,77]. However, marked mast cell adhesion on OP9-Dll1 was not a consequence of the activation of canonical Notch signaling in OP9-Dll1 cells or mast cells. Metabolically inactive mast cells were still capable of effectively tethering to OP9-Dll1 (Figure 3). Marked adhesion was only reversed when the Notch receptor-Dll1 interaction was inhibited by soluble DLL1 as an antagonist or the combined addition of antibodies against Notch1 or Notch2, both of which are expressed on mast cells (Figure 3). Thus, we concluded that Notch1 and Notch2 on mast cells cooperatively function as cell adhesion molecules via Dll1 on stromal cells, leading to the marked promotion of mast cell adhesion.

By using the same strategy as that with other stromal cells transduced with each Notch ligand gene (OP9-Dll1, -Dll3, -Dll4, -Jagged1, and -Jagged2) [78], we found that, similar to the fly DSL ligands, the function of cell adhesion was conserved in the Delta-like (Dll1 and Dll4) and Jagged (Jagged1 and Jagged2) families in mammals [15]. Dll3 did not function in mast cell adhesion, which is consistent with previous findings showing that Dll3 cannot bind to Notch receptors on neighboring cells [38,39].

Previous studies suggested the involvement of vertebrate Notch family members in cell-cell adhesion. A mouse pro-B cell line, which hardly adheres to Chinese Hamster ovary (CHO) cells, effectively anchored to CHO cells expressing mouse Dll1, Jagged1, or Jagged2 [36,51]; however, the contribution of Notch signaling was not determined. Although the overexpression of zebrafish DeltaD in human keratinocytes promoted their cohesiveness, the Notch receptor-ligand interaction did not appear to be required in this case and DeltaD ICD mediated cohesiveness [79,80,81].

## 4. Notch Family Members Mediate Long-Lasting Cell-Cell Adhesion

**Figure 4:**
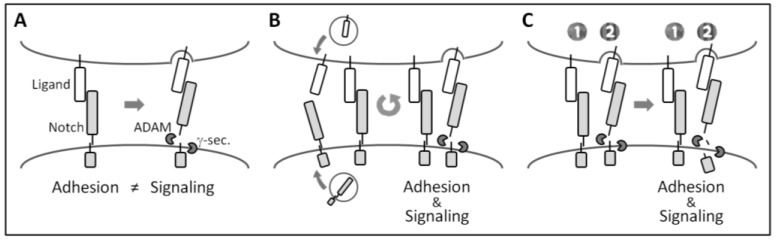
Possible explanations for long-lasting Notch-mediated cell adhesion accompanied by Notch signaling activation in mammals. (**A**) Although adhesion and signaling functions are traditionally considered to be incompatible, our findings suggest that they are compatible; (**B**) the turnover hypothesis. The function of adhesion is transient, consistent with the traditional view. However, newly-formed Notch and DSL ligands successively form Notch receptor-ligand interactions, and, thus, cells appear to stably adhere to each other; and (**C**) the dissociation-resistant interaction hypothesis. There are two forms of Notch receptor-ligand interactions, *i.e.*, dissociation-resistant (1) and dissociation-sensitive (2). The former assumes long-lasting cell adhesion, while the latter assumes signaling activation.

Since the Notch ECD was shown to be immediately dissociated (mammals) or cleaved by ADAMs (flies) after ligand binding [36,68], the adhesion and signaling functions of Notch are incompatible at the single molecular level (Figure 4A). Cell aggregation between fly S2-*d*Notch and S2-Delta cells, and anchoring of the mouse B cell line to CHO-Dll1 cells, were previously reported to occur in a transient manner [51,68], suggesting not only that Notch-mediated cell adhesion is a transient phenomenon, but also that these two functions are incompatible even at the cellular level.

However, we found that marked mast cell adhesion to OP9-Dll1 persisted for days after the co-cultivation. The expression of Notch target genes such as *Hes1* and *Hey1* was increased in adherent mast cells in a manner that depended on γ-secretase activity, indicating that Notch receptors on adherent mast cells are dissociated and cleaved [15]. Thus, our findings suggest not only that the adhesion and signaling functions of mammalian Notch are compatible, even at the cellular level, but also that Notch family members have the ability to mediate long-lasting cell-cell adhesion.

Two possibilities have been suggested as the mechanism responsible for long-lasting Notch-mediated cell adhesion with the activation of Notch signaling. All of the mammalian Notch receptor-ligand interaction transiently mediates cell adhesion until the immediate dissociation of Notch ECD by the endocytic activity of ligand cells; however, newly-synthesized Notch and DSL ligands are successively supplied to the cell membrane and cells appear to stably adhere to each other (Figure 4B). Alternatively, there are two forms of Notch receptor-ligand interactions: a dissociation-resistant interaction that mediates long-lasting cell adhesion and a dissociation-sensitive interaction that mediates signaling (Figure 4C).

Since the pulling force produced by the endocytic activity of ligand cells has been suggested to dissociate the heterodimerization of mammalian Notch at the ECD before ADAM cleavage [65], dissociation resistance may be explained by the inhibition of local endocytosis at some Notch receptor-ligand interaction or the involvement of unprocessed Notch receptors. Although most mammalian Notch receptors are furin-processed heterodimers, a small number of unprocessed full-length Notch receptors are present in cultured cells and tissues [82]. Unprocessed Notch was previously shown to be resistant to dissociation and ADAM cleavage upon ligand binding [65].

The hypothesis that Notch may have the ability to form long-lasting cell adhesion as well as transient adhesion will be an important concept for understanding Notch-mediated cellular responses.

## 5. *In Vivo* Roles of Notch-Mediated Cell Adhesion in Mammals

The most important issue in this field of research is that there has been no conclusive evidence to demonstrate that Notch family members function as cell adhesion molecules *in vivo*. Moreover, since previous studies including ours, which have shown the cell adhesion function, rely on cultured cell lines overexpressing Notch family members, there is a concern that it is merely an artificial phenomenon. Notch-mediated cell adhesion has also been suggested to be a by-product of the strong Notch receptor-ligand interaction in order to induce a conformational change in or the dissociation of Notch ECD by the pulling force. However, mammalian studies have started to suggest that cell adhesion may have *in vivo* roles.

*O*-fucosylation by the Pofut1 enzyme is essential for ligand binding by mammalian Notch receptors [83,84]. Wang *et al.* recently reported that the conditional deletion of *Pofut1*, as well as the administration of neutralizing antibodies against Dll4 or Jagged1 in mice, led to the egress of hematopoietic stem cells (HSCs) from the bone marrow niche cells expressing these ligands [85]. In contrast, the conditional deletion of CSL (RBP-Jκ) resulted in only the modest egress of HSCs. These findings suggest that Notch family members may mediate HSC niche retention as cell adhesion molecules.

Cell adhesion molecules play pivotal roles in the recruitment and localization of immune cells in inflamed tissues [86,87,88] or cancer metastasis [89,90]. Various inflammatory tissues are accompanied by the massive infiltration of immune cells as well as increases in the expression of DSL ligands in endothelial cells and stromal cells [91,92,93,94,95,96,97,98,99]. The systemic inhibition of the Notch receptor-ligand interaction has been shown to reduce the accumulation of several kinds of immune cells [98,100,101,102,103]. Moreover, intestinal mast cells require the expression of Notch2 in order to localize to the epithelium [104]. In pancreatic cancer, the NOTCH1-JAGGED2 interaction itself, but not canonical Notch signaling, is known to promote metastasis [105].

Although the involvement of Notch signaling, particularly non-canonical signaling, in the above processes currently remains unclear, these findings imply that Notch-mediated cell adhesion is utilized by various cell lineages in order to mediate *in vivo* responses in mammals.

## 6. What is the Original Role of Notch-Mediated Cell Adhesion?

The above findings provide examples of the specific incorporation of the cell adhesion function of Notch family members into the cellular responses that emerged during vertebrate evolution. However, the ancestral role of Notch-mediated cell adhesion and evolution of the cell adhesion function have not yet been elucidated. In this section, we discuss the origin of Notch-mediated cell adhesion dating back to metazoan evolution.

### 6.1. Bilateria

**Figure 5:**
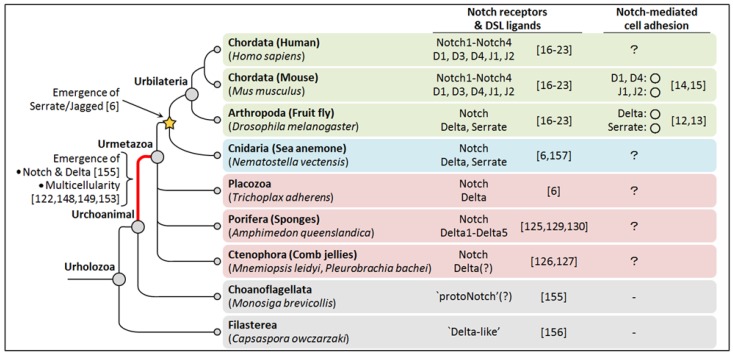
A summary of Notch-mediated cell adhesion in organisms described in this review. The simplified phylogenic tree is depicted based on reference [152]. The last common ancestors of each group are indicated as filled circles at nodes. Branches are collapsed at the base of Metazoa to reflect current uncertainty about the branch order of the most basal metazoan phyla [152]. In Ctenophora, *P. bachei* has the orthologue of Delta [127], while *M. leidyi* has no diagnostic domains for Delta [126]. In the column for Notch-mediated cell adhesion, the existence of the cell adhesion function in Notch family members is shown as open circles. D, Delta-like; J, Jagged.

It currently remains unknown whether it is possible to determine the original role of Notch-mediated cell adhesion by studying bilaterian model organisms. The cell adhesion function of Notch family members in the fly (Arthropoda) and mouse (Chordata) suggests that this function was evolutionally conserved throughout the divergence of these phyla from the hypothetical ancestral animals of Bilateria, called Urbilateria, which lived in the late Precambrian ocean [106,107,108] (Figure 5). Thus, Notch-mediated cell adhesion may have already been present in Urbilateria. This logic and the marked conservation of the domain architecture of Notch family members [6] suggest that the cell adhesion function was a common feature in diverse bilaterian organisms.

Given that the equivalent function of orthologous genes may reflect the past adaptation of ancestral animals and persisting strong selective pressure, namely, loss has a deleterious impact, it is tempting to speculate that there must be some common developmental process(es) in Bilateria that are highly dependent on Notch-mediated cell adhesion. Urbilateria is considered to already have a segmented body, centralized nervous system, and blood circulatory system [106,107,108]. Mice lacking *Pofut1* are embryonic lethal at ~E9.5 with severe defects in somitogenesis, neurogenesis, and cardiovasculogenesis [109,110]. These findings suggest that Notch-mediated cell adhesion evolved to mediate these processes. However, defective organogenesis in mice lacking *Pofut1* is very similar to that in mice with a defect in canonical Notch signaling [109,110,111,112,113], suggesting that the developmental roles of Notch-mediated cell adhesion, if any, are masked by the critical effects of Notch signaling [114,115,116]. Moreover, the requirements of Notch family members during early embryogenesis differ among bilaterian organisms [109,110,113,117,118,119,120,121], raising the question of whether Notch family members were independently integrated into different developmental processes by each species during their divergence [110].

Therefore, it is difficult to obtain a consensus view on the ancestral role of Notch-mediated cell adhesion from extant bilaterian organisms. Furthermore, Notch-mediated cell adhesion in bilaterian organisms may be an inherited feature from more primitive ancestors already possessing Notch family members.

### 6.2. Early-Branching Metazoan Organisms

Extant early-branching metazoan organisms (Ctenophora “comb jellies”, Porifera “sponges”, and Placozoa [122,123,124]) may be the key to understanding the ancestral developmental role of Notch-mediated cell adhesion because they have orthologues of Notch and Delta (but not Serrate/Jagged) despite their simpler body plans [5,6,7,125,126,127,128] (Figure 5).

Notch receptors in Porifera (*Amphimedon queenslandica*) and Placozoa have many of the domains found in bilaterian Notch receptors [6]. Delta ligands in the sponge, *A. queenslandica* already have the MNNL domain with conserved cysteine residues, the DSL domain, and EGF-like repeats, all of which are required for strong receptor-binding in bilaterian Delta [45,53,54,55,56,57,58]. Therefore, Notch and Delta in early-branching metazoans may have already possessed the function of cell adhesion (although we have no information on the domain architecture of Notch and Delta in Ctenophora, which may be sisters to all other metazoan organisms [123,124,126,127]). Since complete Serrate/Jagged is absent in early-branching metazoans [6], the Delta-type ligand is ancestral, while the Serrate/Jagged-type ligand was added later in the Cnidaria + Bilateria stem lineage by the duplication and domain shuffling of ancestral Delta [6,7] (Figure 5). Therefore, the Serrate/Jagged ligand may have inherited the cell adhesion function from ancestral Delta at its emergence.

One Notch and five Delta orthologues in *A. queenslandica* have dynamic and complex expression patterns during its embryogenesis [129,130], suggesting that they have some developmental roles. Cell adhesion plays vital roles in cell segregation and boundary formation between distinct cell populations in order to derive tissue morphogenesis through the mediation of cell sorting and cell migration [131,132,133,134,135,136,137,138]. Gastrulation(-like) embryogenesis in Ctenophora and Porifera was previously shown to be accompanied by active cell compartmentalization and dynamic cell movements [139,140,141,142,143,144,145]. Therefore, we speculate that one of the ancestral roles of Notch-mediated cell adhesion may be tissue separation in order to generate proper morphogenesis during the gastrulation-like embryogenesis of the last common ancestor of Metazoa, called Urmetazoa [146].

However, the emergence of a functional interaction between Notch and Delta may have predated the evolution of the gastrulation-like embryogenesis of Urmetazoa [147,148,149].

### 6.3. Urmetazoa

Urmetazoa has been estimated to arise nearly 800 million years ago [150] and is considered to share the last common unicellular ancestor (*i.e.*, Urchoanimal) with the extant unicellular and colony-forming organisms in Choanoflagellata, which are currently the closest known relatives of Metazoa [151,152,153,154] (Figure 5). Thus, choanoflagellates and their unicellular relatives must hold important clues to the origin of Notch-mediated cell adhesion.

The genome of the choanoflagellate *Monosiga brevicollis* has shed light on how Notch evolved [155]. *M. brevicollis* has no *bona fide* Notch, but possesses three genes that encode each protein domain (EGF-like repeats, LNRs, and Ankyrin repeats) found in metazoan Notch receptors. Therefore, the *bona fide* Notch receptor with the ability to activate the canonical signaling cascade may have emerged by domain shuffling at some point during the evolution of Urmetazoa [155]. One of the above three genes in *M. brevicollis*, which has tandem EGF-like repeats and a transmembrane domain [155], is of particular interest because it suggests that a membrane-bound molecule similar to the ECD of metazoan Notch may already be present in the Urchoanimal (hereafter we call this gene “protoNotch” for convenience, assuming that a similar gene existed in Urchoanimal).

The Delta ligand itself and even the MNNL and DSL domains are absent from the *M. brevicollis* genome [155,156]. However, a “Delta-like” receptor protein tyrosine kinase, which has two DSL domains and tandem EGF-like repeats in its ECD, has been found in the genome of the Filasterea *Capsaspora owczarzaki*, another unicellular close relative of Metazoa [156]. It currently remains unclear whether this gene was more ancestral or a specific invention by *C. owczarzaki*. These findings indicate that the DSL domain was present in Urchoanimal, and the Delta ligand may have evolved through a combination of an already existing DSL domain and EGF-like repeats, in addition to the invention and incorporation of the MNNL domain [6], at some point during the evolution of Urmetazoa.

Since *M. brevicollis* has ADAMs, the γ-secretase complex, CSL, and many of the other Notch pathway components found in early-branching metazoans [6,155,156], the functional Notch signaling pathway may have been formed by the incorporation of already existing components downstream of the newly-emerged Notch-Delta interaction. It is difficult to determine when and in what order the following revolutions occurred; (1) the Delta ligand emerged; (2) the protoNotch gained its functional ICD with several domains to become *bona fide* Notch with the ability to signal; and (3) the complex Notch signaling cascade began functioning. However, since the completion of the Notch signaling pathway must pass through multiple molecular revolutions that enable functional interactions between each downstream component and functional incorporation into cellular responses in order to regulate gene expression, we speculate that the emergence of the protoNotch-Delta interaction itself antedated the completion of the signaling function.

If the protoNotch-Delta interaction itself did not have any functions, it is unlikely that the association of these molecules was positively selected and maintained until the signaling pathway was completed during Urmetazoa evolution. Multicellularity is considered to give ancestors distinctive advantages in escaping from predators, establishing a new colony, or enhancing the capture of prey [122,149,153]. If the protoNotch-Delta interaction had the cell adhesion function and contributed to stable intercellular adhesion in order to support the early form of multicellularity, this may explain why these molecules were positively selected and maintained during the evolution of Urmetazoa before the completion of its signaling function.

Therefore, we hypothesize that Notch family members initially arose as cell adhesion molecules that played a role in the formation of early metazoan multicellularity. This may be the origin of Notch-mediated cell adhesion.

## 7. Conclusions

The origin of Notch-mediated cell adhesion may date back to the emergence of metazoan multicellularity and Notch family members may have arose as cell adhesion molecules. It is interesting to note that cell adhesion molecules such as cadherins, integrins, and some extracellular matrices (ECMs), or several domains of metazoan-type ECMs, which were once recognized as specific innovations of Metazoa, have been found outside Metazoa [155,158,159,160,161,162,163,164,165]. Classical cadherins and metazoan-type ECMs such as fibrillar collagens appear to have a truly metazoan origin, and, thus, are regarded as strong candidates for contributing to the evolution of multicellularity [163,164,165,166]; however, it currently remains unknown whether they were present in Urmetazoa before the emergence of Notch family members. Therefore, along with these molecules, Notch family members represent an appealing candidate as the driving force for metazoan multicellularity.

Notch-mediated cell adhesion may have subsequently been incorporated into novel developmental and cellular processes that emerged during metazoan evolution and now possibly control HSC niche retention, immune cell recruitment, and localization in the human body. Therefore, Notch-mediated cell adhesion will be important as a therapeutic target for stem cell therapy, inflammatory disorders in order to prevent the undesired accumulation of immune cells, and also cancer metastasis.

We are only beginning to understand the mechanisms of and the cellular processes regulated by Notch-mediated cell adhesion. Clearly, we require more significant experimental evidence which explains the molecular basis of these processes. We anticipate that, the old, but generally unaccepted appreciation that Notch family members are cell adhesion molecules as well as signaling molecules will provide a new perspective for understanding the broad spectrum of Notch-mediated cellular responses in Metazoa.
